# The efficacy of the enhanced Aussie Optimism Positive Thinking Skills Program in improving social and emotional learning in middle childhood

**DOI:** 10.3389/fpsyg.2014.00909

**Published:** 2014-08-15

**Authors:** Jacqueline D. Myles-Pallister, Sharinaz Hassan, Rosanna M. Rooney, Robert T. Kane

**Affiliations:** Faculty of Health Sciences, School of Psychology and Speech Pathology, Curtin UniversityPerth, WA, Australia

**Keywords:** middle childhood, social emotional learning, universal school-based program, Aussie Optimism

## Abstract

The aim of the current study was to investigate the effects of the modified and enhanced *Aussie Optimism Positive Thinking Skills Program* (AO-PTS) on Year 4 and 5 children's social and emotional learning (SEL) skills. AO-PTS is a universal-school based program that is implemented by class teachers as part of regular school curricula and was developed for the prevention of depression and anxiety. The study comprised a total of 683 Year 4 and 5 students from 10 private primary schools in Western Australia. Students were assessed on two subscales of emotional attribution at school whilst parents reported on their children's externalizing and internalizing problems outside of school and at home. Two analyses were conducted: seven intervention schools were assessed at pre- and post-test (Analysis 1) and pre-post change in three intervention schools were compared to pre-post change in three matched control schools (Analysis 2). Results from Analysis 1 showed that the intervention children had increased in their overall emotional attribution accuracy and decreased in total difficulties and hyperactivity; Results from Analysis 2 revealed no intervention effect on emotional attribution accuracy or internalizing or externalizing problems. These findings suggest that the enhanced AO-PTS's effects on SEL were not evident in the short-term period after intervention. The non-significant findings and future directions for AO-PTS research and program modification were discussed.

## Introduction

Well-developed social and emotional skills in childhood provide resilience for both the home and school environment and in later life (Zins and Elias, 2006; Payton et al., 2008). An ability to negotiate social relationships and have good awareness of one's own and others' emotions provides a backdrop onto which children can either thrive in and outside of school or alternatively that they can be faced with a host of potential problems (Bernard et al., 2007; Denham and Brown, 2010). Well-developed social and emotional skills are related to good academic outcomes; conversely, children with poorly developed emotional skills may be more likely to have behavioral adjustment problems, more sexual partners, and are more likely to use hard drugs (Denham and Brown, 2010; Hessler and Katz, 2010). Barblett and Maloney (2010) highlighted that the “empirical research is overwhelming in providing evidence that strong growth in social and emotional competence and wellbeing underlies all later growth and development” (p. 14).

Social and emotional skills are important for all children, not just those at-risk. A recent report on the social and emotional health of Australian children reported a steady decline in students' ratings of their social and emotional well-being that began in the middle years of primary school (Bernard et al., 2007). In addition the ratings by students of their social and emotional well-being did not differ between those in the highest and those in the lowest SES ranges. This indicates that social and emotional health and the skills that promote these are relevant to children from all walks of life.

Social and emotional learning (SEL) is the process of acquiring the knowledge, attitudes and skills that permit children to identify and cope with their emotions, set and work toward goals, be able to empathize with others, make decisions and be able to effectively navigate interpersonal relationships, including keeping and maintaining friendships (Payton et al., 2008). SEL capabilities are inextricably linked with a child's social and emotional well-being (AIHW, 2012). Denham and Brown (2010) outline five core SEL competencies: (1) self-awareness; (2) self-management; (3) social awareness; (4) responsible decision making; and (5) relationship/social skills. These essential skills allow children to regulate their emotions when they get angry or distressed, start up and continue relationships with their peers and other people, and deal with challenging situations when they arise.

A considerable amount of literature has been published on SEL universal programs and point to a range of positive benefits for children. These benefits are demonstrated by the findings from two recent meta-analyses of universal school-based SEL programs. One meta-analysis was conducted on 213 school-based universal SEL programs that included 270, 034 kindergarten through to high school students (Durlak et al., 2011) and the second meta-analysis was conducted on 180 studies (including 277, 977 kindergarten through to Year 8 students; Payton et al., 2008). These two meta-analyses indicate that SEL programs enhanced students' behavioral adjustment in the form of increased prosocial behaviors (more positive social behaviors, prosocial attitudes toward aggression, more positive attitudes toward self and others); reduced conduct problems; reduced internalizing problems (lower levels of emotional distress in the form of anxiety and depressive symptoms), and improved academic performance (with an 11 point percentile increase in academic achievement test scores) (Payton et al., 2008; Durlak et al., 2011). Effect sizes of the universal SEL programs ranged from 0.22 to 0.57 (Durlak et al., 2011). In addition, the findings indicated that school teachers and other school staff can effectively conduct SEL programs in schools; in fact the effect sizes for outcomes relating to attitudes, positive social behavior, conduct problems, emotional distress, and academic performance were better when teachers ran the programs, compared to when non-school personnel conducted the programs (Durlak et al., 2011). The authors of both reviews concluded that universal school-based SEL programs can be effective and have a positive impact on multiple areas of students' lives.

More recently, many universal school-based SEL studies have been conducted to investigate the impact of SEL programs on children. For instance, one large scale randomized clinical trial evaluated an adaption of the Promoting Alternative Thinking Strategies curriculum (PATHS) on pre-school age children. After receiving the PATHS program, the students' in the 10 intervention classrooms were rated by their parents and teachers as more socially competent, less socially withdrawn and had higher emotion knowledge skills, compared to students in the control classrooms (Domitrovich et al., 2007). Another PATHS study, which was a longitudinal evaluation of children followed from Grade 1 to Grade 3, found a reduction in teacher and peer reported aggression and an increase in prosocial behavior, as well as teacher-reported improvements in students' academic engagement (Conduct Problems Prevention Research Group, 2010). A study of *Head Start—*an emotion-based prevention program in 2- to 5-year old rural and urban children—reported greater increases in emotion knowledge, emotion regulation, positive emotion expression, and social competence (Izard et al., 2008). Further evidence comes from studies on children in middle primary school. For instance, the “Roots of Empathy” program was implemented on 4th- to 7th-grade children and reported improvements in prosocial behavior, proactive and reactive aggression, however, empathy and perspective taking did not show improvements (Schonert-Reichl et al., 2012). Collectively, these findings indicate that universal school-based SEL programs are promising for improving children's social and emotional competence. However, little research has been conducted on children in middle primary school and it is not known if depression and anxiety prevention universal school-based programs can also positively impact on children's SEL skills.

Another universal school-based program is the Aussie Optimism Program: Positive Thinking Skills (AO-PTS). Although AO-PTS was originally developed as a program to prevent depression and anxiety in children in middle childhood, the contents of the program also fit well with core aspects of SEL programs. Many SEL programs are long in duration (e.g., 25 lessons and three years, respectively, Conduct Problems Prevention Research Group, 2010; Schonert-Reichl et al., 2012) or target specific behaviors such as aggression (Conduct Problems Prevention Research Group, 2010). In contrast, AO-PTS is delivered by school teachers as part of the regular curriculum over 10 weekly sessions. Furthermore, the contents of AO-PTS are consistent with the five core interrelated factors, as outlined by Collaborative for Academic, Social, and Emotional Learning (Collaborative for Academic Social and Emotional Learning, 2005), that SEL programs need to address, including: accurately assessing one's feelings, regulating emotions and working toward goals, being able to empathize with others, establishing and maintaining healthy and rewarding relationships and making responsible decisions.

Research conducted on Aussie Optimism Programs (AOP) aimed at older children indicate that AOP may have some impact on children's SEL outcomes, however, the findings have been mixed. For instance, these studies have reported reduced parent reported internalizing symptoms in Grade 7 students from disadvantaged schools, but not for externalizing or social skills (Roberts et al., 2010); reduced total difficulties and conduct problems in Grade 8 students (Swannell et al., 2009); and reduced likelihood of using tobacco and alcohol at the end of Grade 8 when the children received AOP in Grade 6 and 7, but only when the teachers implementing the program received coaching (Roberts et al., 2011). These findings have been somewhat inconsistent and tell us little about AOP impact on SEL for younger children.

To date, five studies have been conducted on the AO-PTS. A pilot study conducted on 72 8- to 9-year old children from four low socioeconomic (SES) state primary schools, who were randomly allocated to either intervention or health education as usual, found lower levels of depression and more positive attribution styles in intervention children, compared to control children (Rooney et al., 2006). In addition, less of the intervention children maintained a diagnosis of depression at post-test or developed a depressive disorder at 9-month follow-up, compared to control children (Rooney et al., 2006). Similarly, a large scale randomized trial of AO-PTS, conducted on 910 Year 4 and 5 children from 22 low SES schools, also found that intervention children were significantly less depressed at post-test as well as less parent-reported total difficulties at post-test and 6 months following the intervention when compared to control children (Rooney et al., 2013). Both intervention and control children increased in parent-reported prosocial behaviors at post-test, 6- and 18-month follow-up assessments. Conversely, no significant differences were found between the children who had received AO-PTS and those who received regular health education on outcomes relating to hyperactivity, conduct or peer problems (Rooney et al., 2013). A 30-month follow-up study was conducted with the same cohort of students to examine the medium-term effect of AO-PTS. Results showed that there was a significant reduction in hyperactive behaviors for the intervention children; however, no significant differences were found in the depressive, anxiety and attribution styles across groups (Morrison et al., 2013). Furthermore, two follow-up studies conducted at 42- and 54-months post intervention, aimed at determining the longitudinal effect of the AO-PTS, found no significant reduction in depression, anxiety and attribution styles across groups (Johnstone et al., 2014). The AO-PTS has now been enhanced to include more content on social and emotional competence and a less complex cognitive component which is more developmentally appropriate (Rooney et al., 2009). A small pilot study with the enhanced version of the AO-PTS was conducted in a rural setting and found that control group children had significantly increased in the parent-reported levels of peer problems at post-test when compared to the intervention children (Lacey et al., submitted).

In summary, research on universal school-based programs has found enhanced behavioral adjustment, with increased prosocial behavior and academic performance, and reduced conduct and peer problems; however, many of these programs involve long implementation periods, target certain behaviors, and little is known about the effects of depression and anxiety prevention programs on children's SEL skills. Research on older children using AOP programs have generally been conducted over 20 sessions and have reported mixed findings related to SEL factors. Although there have been a number of studies conducted on AO-PTS, particularly with relation to its effect on depression and anxiety, little research to date has reported on the effects of the enhanced version of AO-PTS on children's social and emotional skills.

To address these identified gaps in the literature, given that there is a decline in children's self-reported social and emotional well-being beginning in middle childhood, the present study aimed to investigate the impact of the enhanced AO-PTS program on the social and emotional development of Years 4 and 5 children. Private schools were chosen as there is a paucity of research on students in these schools and evidence suggests that aspects of these schools may present challenges to the mental health of children who attend them (Watt, 2003). In addition, children from both high and low SES do not differ in these subjective reports of well-being. The implications of positive findings would be that there is a universal school-based program that can be successfully implemented by teachers as part of the regular school curriculum, which not only has strong evidence for its preventative effects on depression, but also positively impacts on children's general SEL skills, with the potential to benefit many areas of their home and school life. To this end, the following hypotheses are proposed: At post-test, parents of students in the intervention group will report lower levels of internalizing and externalizing symptoms and higher levels of prosocial behaviors and lower levels of total difficulties in their children (as measured by the SDQ-P) than parents of students in the control group (Hypothesis 1). At post-test, the intervention group will report higher levels of emotional attribution accuracy (as measured by the ACES) than the control group (Hypothesis 2).

## Methods

### Participants

Participants were 683 Years 4 and 5 students from 10 private primary schools in Western Australia, which represents 56.63% of the available 1206 Year 4 and 5 in these schools. There were approximately equal numbers of males and females (350 boys [51.2%] and 333 girls [48.6%]). At pre-test, participants were aged between 8.75 and 11.58 years. The large age range reflects the fact that some children had their pre-test in 2010, some in 2011, and others in 2012. A breakdown of the children's ethnicity is provided in Table S1.

The gender and number of students in each year group in both the intervention and control conditions are presented in Table S2. Students in the intervention group received the AO-PTS program delivered by their teachers as part of their normal school curriculum; students in the control condition received their regular health education lessons.

This study used a sub-set of data from an ongoing larger AO-PTS research which will collect data from 30 private schools which have been randomly selected from a range of socioeconomic areas in Western Australia. SEL universal interventions have been found to be equally beneficial for both socioeconomically advantaged and disadvantaged students (Trentacosta and Fine, 2009). To date, data for Year 4 and Year 5 students from seven intervention schools and three control schools were available and this is the data used in the current study.

### Research design

For the overarching study, before pretesting, 30 private schools were randomly allocated to one of two conditions—school-based intervention or control—with 15 schools in each condition. This research study used a nested cohort design (Murray and Hannan, 1990): schools were allocated to either the intervention or control condition and the students were then followed as a cohort to determine the effects of the intervention. This research design consisted of two categorical random effects (school and student), one categorical fixed effect (group: intervention, control), and one ordinal fixed effect (time: pre, post). This design generated a hierarchical data structure in which time is nested within student and student is nested within school. There are two families of outcome variables: (i) self-reported emotional knowledge and recognition (measured by ACES), (ii) parent evaluations of the internalizing and externalizing problems which include the prosocial behavior and total difficulties, in their child (measured by SDQ-P).

### Instruments

The *Assessment of Children's Emotional Skills* (ACES; Schultz et al., submitted) was used to assess children's self-reported emotional knowledge and emotional recognition ACES includes three sections that reflect the child's accuracy of emotion attribution relating to social behaviors, social situations, and facial expressions (Schultz et al.). This study used the social behaviors and social situations subscales. Each of these sub-scales included 15 one- to three- sentence items to which the child labeled the portrayed character's feeling as “happy,” “sad,” “mad,” “scared,” or “no feeling.” Children received a score of either 1 (correct) or 0 (incorrect) for each item. Three items were related to each of the emotions under each subscale. Twelve of the 15 items on each subscale, excluding the ambiguous (“no feeling”) items, were summed to produce a total score for each of the subscales yielding a score out of 12; the total scores of these the two subscales were summed to produce the ACES total score. The scores for emotional assessment range from 0 to 24, with high scores indicating greater emotional attribution accuracy. The reliability for the three subscales (facial expression, social behavior, and situations) is moderately high (*r* = 0.68; Schultz et al.). The internal consistency coefficient for the total emotion accuracy scale with the current sample at pre-test was moderate (α = 0.61); internal consistency coefficients for the social situation and social behavior subscales were both low (α = 0.47) and ranged between 0.16 and 0.43 for the emotion subscales that constituted these two subscales. Thus, all three of these scales, which included 10 or more items, did not obtain a Cronbach's alpha of 0.7+ indicating good internal consistency (Tabachnick and Fidell, 2007). In addition, the subscales of the social behaviors and social situations, which contained less than 10 items each, did not meet a sufficient Cronbach's alpha of around 0.6 (Loewenthal, 2001). The test-retest reliability in the current study for the Total Emotion Attribution Score was moderate (*r* = 0.56) and for the Behavioral Situations and Social Situations were 0.42 and 0.52, respectively. In addition, the ACES has been found to correlate well with trait emotional intelligence (Mavroveli et al., 2009).

The parent version of the *Strengths and Difficulties Questionnaire* (SDQ-P; Goodman, 1999) was used to assess children's overall psychological adjustment and provide a measure of social and emotional competence (see Supplementary Material). The parent version of the SDQ-P measures internalizing and externalizing problems in the home, designed for use by parents/caregivers of children 4–16 years. There are 25 items measuring internalizing and externalizing problems across five subscales—Emotion Symptoms, Conduct Problems, Hyperactivity/Inattention, Peer Difficulties, and Prosocial Behavior. A Total Difficulties Score is computed by summing across the first four subscales. Parents rate each given item as either “not true,” “somewhat true,” or “certainly true.” Internal consistency coefficients range between 0.76 and 0.82 for the total and summative scores. The internal consistency coefficient for the current sample at pre-test for the Total Difficulties Score was high (α = 0.84) and ranged between 0.64 and 0.82 for the five subscales, indicating sufficient or good internal consistency (Loewenthal, 2001; Tabachnick and Fidell, 2007). The SDQ-P has strong 2-week test-retest reliability (0.96 for the total score; Goodman, 1999). The test-retest reliability in the current study was very high for the Total Difficulties Score (*r* = 0.92) and ranged between 0.73 and 0.82 on the five subscales. The instrument correlates well with the Rutter Parent Scale and the Child Behavior Checklist (Goodman and Scott, 1999). Numerous studies have found the SDQ to have good concurrent and discriminant validity in non-clinical samples (Goodman, 1999; Goodman and Scott, 1999; Muris et al., 2003).

*A Demographic questionnaire* was used to collect background demographic information from parents regarding: socioeconomic status (SES), ethnic origin, family structure, family health, history of mental health problems, and contact details.

### Procedure

This study used a subset of available data from a larger on-going Aussie Optimism Positive Thinking Skills project. The procedure for the larger project involved the selection of thirty primary schools from all private schools in the Perth Metropolitan area who were assigned at random to either the intervention or control condition. School principals were invited to participate with the understanding that assessments would take place at both pre- and post-tests. The larger project researchers will be collecting 6- and 18-month follow-up data, whereas the current study used the pre- and post-test data from seven intervention schools and three control schools only. The teachers from participating intervention schools attended an 8 h training course in which they were taught the principles of the Positive Thinking Skills program and how to effectively run groups.

The enhanced *Aussie Optimism Program: Positive Thinking Skills Program* (AO-PTS; Rooney et al., 2009) is a universal school-based program that involves 10 1-h weekly sessions which are delivered as part of the school curriculum. The 10 sessions target areas such as the identification and awareness of comfortable and uncomfortable feelings, understanding feelings in others, cognitive re-structuring, a fear hierarchy, pleasant events scheduling and weekly relaxation training. In later modules, there is a focus on situations relevant to peer and family relationships. Each of the 10 modules has specific learning outcomes and teaching strategies, such as teacher-led group discussions, role plays, and skills practice sessions. There are three program booklets in the enhanced AO-PTS program: a teacher resource manual (Teacher Resource: Aussie Optimism Positive Thinking Skills; Rooney et al., 2009), a student workbook (Nesa et al., 2004), and a self-directed parent manual (Nesa and Rooney, 2004). Table S3 outlines the topics and aims of each of the 10 modules in the teachers and student workbooks.

The intervention schools and teachers were offered 5 × 1 h coaching sessions. The active consent of children and parents was sought after the study has been fully explained to them via school meetings and a formal information and consent form was sent home. The assessment battery was administered to the children by trained research assistants to provide baseline pre- and post-test measures of emotional competency. Measures for the parents were sent home through the school in sealed envelopes at pre- and post-test. The weekly intervention sessions were run in the usual class room groups.

The groups were conducted by teachers at the school at convenient designated times over 10 weekly 1-h sessions. Although monitoring of adherence to the assigned interventions was planned using implementation checklists, log books, reports, interviews, and observations by research assistants, resistance was received by teachers regarding filling-in the integrity check-lists and having their sessions observed. One possible reason for teachers' reluctance to complete the integrity check-lists is the potential extra workload. Integrity check-lists required the individual assessment of attendance and level of participation in each students' log-book. Hence, with the risk of many schools withdrawing from the study, these aspects of the project were dropped.

### Data analysis

The aim of the present paper was to determine whether there were intervention effects at the post-intervention assessment. These data were analyzed with a multi-level mixed effects linear regression model (Bryk and Raudenbush, 1987) as implemented through SPSS's Generalized Linear Mixed Models (GLMM: SPSS Version 19) procedure. In order to test the relationships between the fixed effects and the outcomes, GLMM assumed a normal probability distribution for each outcome and linked it to the fixed effects with an identity function. If the outcomes did not have a normal distribution, then the parameter estimates of the covariance matrix were computed with robust statistics. Finally, in order to optimize the likelihood of convergence, separate GLMM analyses were run for each of 13 student-reported ACES outcomes (Total Emotion Attribution, Total Behavior and Social Situations Attribution, with related five feelings subscales) and for each of the parent-reported SDQ outcomes, the Total Difficulties (Emotional Symptoms, Conduct Problems, Hyperactivity /Inattention, Peer Relationship Problems) and Prosocial Behavior. As analysing each outcome independently of the others would inflate the familywise error rate, the per-test alpha was corrected to control the inflation. In order to conserve statistical power, the alpha correction *within* groups of conceptually related outcomes, rather than *across* the entire set of outcomes was applied.

### Power

Data from previous school-based intervention studies (e.g., Roberts et al., 2010) were used to estimate the magnitude of these interactions, and the degree to which scores on the outcomes would cluster within-schools. These estimates were input to Murray and Hannan's (1990) formula and then multiplied by the design effect—a number that accounts for the reduction in effective sample size due to intra-class clustering in the data (Campbell et al., 2004). The computation indicated that 12.86 students from each of the seven schools in Analysis 1 (*N* = 196.74) and 15 students from each of the six schools (*N* = 216) in Analysis 2, should give the GLMM an 80% chance of capturing “small to moderate” interaction effects (i.e., *f* = 0.15) at an alpha-level of 0.05. Unlike repeated measures ANOVA (or ANCOVA), GLMM does *not* rely on participants providing data at every assessment point; GLMM uses all the data present at each assessment point thereby reducing the impact of subject attrition on statistical power. Moreover, GLMM is robust to unequal group sizes.

## Results

Two separate analyses were conducted to evaluate the enhanced AO-PTS program as fewer control group data were available than intervention group data. A primary analysis was conducted on seven intervention schools, which consisted of 530 Years 4 and 5 students, to compare the pre-intervention to post-intervention student and parent outcomes (Analysis 1). A secondary analysis was conducted comparing three intervention and three control schools, consisting of 371 Years 4 and 5 students (Analysis 2). The schools were matched in pairs with regards to SES and number of Year 4 and 5 students attending the school.

### Attrition and missing values

Data were screened for accuracy. Missing values due to partially completed questionnaires were replaced with estimates derived from the expectation-maximization algorithm (e.g., Rubin et al., 2007; Graham, 2009).

#### Student attrition

In Analysis 1, the student cohort comprised 471 students at pre-test, of which 458 responded at post-test (a 2.76% attrition rate). Significant differences were found for the means of completers and non-completers on the mean Total Emotion Attribution Score, the mean Total Social Behaviors scores (and the related subscales for the emotions Happy and Mad), as well as on the Total Social Situations scores (and the related subscale for the emotion Scared) (*p*s < 0.05). With the exception of the Happy Social Behaviors subscale, the completers showed higher rates of emotion attribution accuracy compared to the non-completers. In Analysis 2, the student cohort comprised 353 students at pre-test, of which 325 responded at post-test (a 7.93% attrition rate). The rate of attrition between the intervention schools (5.55%) and control schools (19.61%) was significant, χ^2^(1) = 35.07, *p* < 0.001. Significant differences were obtained on the pre-test outcomes between those students who were retained and those who dropped out, such that the completers had significantly higher emotion attribution accuracy on the Total Social Situations subscale, and on the Sad Social Behavior subscale.

#### Parent attrition

In Analysis 1, the parent sample at pre-test comprised 320 parents, of which 191 responded at post-test (a 40.31% attrition rate). There were no significant differences on any of the parent pre-test outcomes between the completers and non-completers. In Analysis 2, the parent sample at pre-test comprised 241 parents, of which 162 responded at post-test (a 32.78% attrition rate). Attrition rates differed significantly between groups—with 43.56% non-completers for the intervention schools and 35.58% non-completers for the control schools, χ^2^(1) = 4.34, *p* = 0.037; however, there were no significant differences on any of the parent pre-test outcomes.

### Student-reported outcome

Table S4 presents the pre- and post-test estimated means and standard errors on the ACES for the interventions schools in Analysis 1, and for the intervention and control schools in Analysis 2. In Analysis 1, there were significant effects of time on children's Total Emotion Attribution Score, *F*_(1, 929)_ = 67.69, *p* < 0.001; Total Social Behavior, *F*_(1, 929)_ = 19.96, *p* < 0.001; and Total Social Situations, *F*_(1, 929)_ = 27.90, *p* < 0.00. Significant time effects were also found for accuracy of attributing Happy Social Behaviors, *F*_(1, 929)_ = 11.64, *p* = 0.001; Mad Social Behaviors, *F*_(1, 929)_ = 26.37, *p* < 0.001; and Ambiguous Social Behaviors, *F*_(1, 929)_ = 340.99, *p* < 0.001; Mad Social Situations, *F*_(1, 929)_ = 7.32, *p* = 0.007; and Scared Social Situations, *F*_(1, 929)_ = 28.06, *p* < 0.001. No significant time effects were found on other Behavior or Social Situation subscales. Table S4 indicates that there was also a trend for improvements in children's ability to accurately attribute emotions in scales that did not reach significance.

In Analysis 2, there was only one significant interaction effect for the Sad Behavioral Situations subscale, *F*_(1, 676)_ = 17.91, *p* < 0.001; LSD *post-hoc*s indicated that, although significant improvements were seen for both the intervention (*p* = 0.002) and control (*p* < 0.001) school children, this improvement was greater in the control schools—indicating no intervention effect. There were significant Time effects found on the Total Emotional Attribution Scale, *F*_(1, 676)_ = 58.66, *p* < 0.001; Total Social Situations, *F*_(1, 676)_ = 10.58, *p* = 0.001; and Total Social Behaviors, *F*_(1, 924)_ = 10.58, *p* = 0.001. There were also significant Time effects found on Sad Social Behaviors, *F*_(1, 676)_ = 71.08, *p* < 0.001; Mad Social Behaviors, *F*_(1, 676)_ = 9.88, *p* = 0.002; Scared Social Behaviors, *F*_(1, 676)_ = 17.78, *p* < 0.001; and Ambiguous Feelings Social Behaviors, *F*_(1, 676)_ = 759.66, *p* < 0.001. Similarly, significant Time effects were observed for Happy Social Situations, *F*_(1, 676)_ = 12.95, *p* < 0.001, and Scared Social Situations, *F*_(1, 676)_ = 13.92, *p* < 0.001. These significant Time effects indicated that both the intervention and control school children had showed improvements in their overall ability to accurately attribute emotions across social behaviors and social situations (see Table S4). There were no other significant times or group effects on the other scales. Once again there was a tendency for outcomes to change positively over time for both groups.

#### Gender differences

In Analysis 1, there was a significant main effect of gender on Emotion Attribution Score, *F*_(1, 895)_ = 11.71, *p* = 0.001; such that females had higher rates of Total Emotion Attribution compared to males at both Time 1 and Time 2. There was no significant Gender^*^Time interaction, *F*_(1, 895)_ = 41.31, *p* = 0.125. In Analysis 2, there was a significant Gender^*^Time interaction, *F*_(1, 661)_ = 10.67, *p* = 0.001. LSD contrasts indicated that males had a significantly greater pre-post increase [Time 1: *M* = 18.62 (*SE* = 0.31) to Time 2: *M* = 19.68 (*SE* = 0.19); *p* < 0.001], compared to females [Time 1: *M* = 19.92 (*SE* = 0.34) to Time 2: *M* = 20.60 (*SE* = 0.28); *p* < 0.001]. The findings also suggest that overall girls across both groups had significantly higher rates of overall Emotion Attribution Accuracy. The Gender^*^Group^*^Time interaction for Total Emotion Attribution Score was not significant, *F*_(1, 661)_ = 0.44, *p* = 0.510, indicating no differential impact of the intervention depending on gender.

#### Grade differences

In Analysis 1, there was a significant Grade effect, *F*_(1, 927)_ = 9.43, *p* = 0.002, such that Year 5 students had significantly greater Emotion Attribution Scores at Time 1 and Time 2, compared to Year 4 students. There was no significant Grade^*^Time interaction on Emotion Attribution Score, *F*_(1, 927)_ = 2.12, *p* = 0.145. In Analysis 2, there was a significant Grade^*^Group^*^Time interaction on Total Emotion Attribution Score, *F*_(1, 372)_ = 7.96, *p* = 0.005. LSD contrasts indicated that for the Year 4s, there was no pre-post change in the intervention group (*p* = 0.056), although this approached significance, but a significant pre-post increase in the control group (*p* < 0.001); for Year 5s, there was a significant pre-post reduction in the intervention group (*p* < 0.001) and an equivalent pre-post reduction in the control group (*p* < 0.001). These results indicate that there were no preferential intervention effects for Year 4s or Year 5s. There was also a significant grade effect, *F*_(1, 672)_ = 7.51, *p* = 0.006, indicating that the Year 5 children had higher emotion attribution accuracy at both Time 1 and Time 2, compared to Year 4 children.

### Parent-reported outcome

In Analysis 1, there were significant effects of time on the SDQ-P Total Difficulties Score, *F*_(1, 510)_ = 9.27, *p* = 0.002, and on Hyperactivity/Inattention Problems, *F*_(1, 510)_ = 8.95, *p* = 0.003, indicating that parents of students who had received the AO-PTS program reported significantly reduced levels of total difficulties and hyperactive/inattentive symptoms in their children at post-test (see Table S5). In contrast, there were no significant Time effects found on Conduct, Peer, and Prosocial subscales (*p*s > 0.05). However, Table S5 shows a trend across all five SDQ-P subscales for improvements in parent-reported scores, with reduced levels of Emotion, Conduct, Hyperactivity, and Peer Problems and increases in Prosocial Behavior.

In Analysis 2, there was only one significant interaction effect on the Peer subscale of the SDQ-P, *F*_(1, 400)_ = 5.28, *p* = 0.022; however, LSD *post-hoc* analyses indicated that this effect was in an unexpected direction, such that parents of intervention children reported significant increases in their children's level of Peer Relationship Problems (*p* = 0.012), whereas there was no significant change in control school children (see Table S5). Significant Time effects were observed for Total Difficulties Scores, *F*_(1, 400)_ = 17.01; *p* = 0.042; as well as on Emotion, *F*_(1, 400)_ = 7.14, *p* = 0.008; and Conduct Problems, *F*_(1, 400)_ = 23.01, *p* < 0.001, such that parent-reported levels of problems in these area significantly reduced in both the intervention and control school children. In addition, there were significant group differences found on Total Difficulties, *F*_(1, 400)_ = 23.07, *p* < 0.001, and Hyperactivity/ Inattention scales. Inspection of the means indicate that control school children had higher levels of both Total Difficulties and Hyperactivity at both pre- and post-test assessments. No other significant group or time effects were observed. Although, the Time effect for Hyperactivity/ Inattention Problems approached significance, *F*_(1, 400)_ = 3.70, *p* = 0.055.

#### Gender differences

In Analysis 1, the Gender^*^Time interaction on the Total Difficulties Score was not significant, *F*_(1, 474)_ = 0.05, *p* = 0.825. The main effect of gender approached significance, *F*_(1, 474)_ = 3.69, *p* = 0.055. In Analysis 2, there was a significant Gender^*^Group^*^Time interaction on The Total Difficulties Scale of the SDQ, *F*_(1, 386)_ = 29.05, *p* < 0.001. LSD comparisons indicated that, for males, there was no pre-post change in the intervention group (*p* = 0.911), but a significant pre-post decrease in the control group (*p* < 0.001). For females, there was a significant pre-post reduction in the intervention group (*p* < 0.001) and control group (*p* = 0.002). These results indicated no preferential outcomes due to the intervention. There was also a significant main effect of gender, *F*_(1, 386)_ = 48.12, *p* < 0.001, and time, *F*_(1, 386)_ = 14.04, *p* < 0.001; a comparison of the mean Total Difficulties indicated that females across both groups had significantly lower Total Difficulties compared to males at pre- and post-test.

#### Grade difference

In Analysis 1, there was no significant Grade^*^Time interaction Scores or Grade effect on Total Difficulties (*p*s > 0.05). In Analysis 2, there was a significant Grade^*^Group^*^Time interaction on Total Difficulties Scores, *F*_(1, 396)_ = 6.00, *p* = 0.015. LSD contrasts found that, for the Year 4 students, there was no pre-post change in the intervention group (*p* = 0.742) but a significant pre-post decrease in the control group (*p* = 0.001); for Year 5 students, there was a significant pre-post reduction in the intervention group (*p* < 0.001) but no pre-post change in control group (*p* = 0.089). These results indicated that the Year 5s, but not the Year 4s, benefitted from the intervention; although, the change in means was small and may not be clinically meaningful and the Year 4 control children also improved.

## Discussion

The aim of the current study was to investigate whether the enhanced AO-PTS, a universal schools-based program developed for the prevention of anxiety and depression would have a positive impact on children's social and emotional competence in middle primary school children. The findings from Analysis 1, provided some encouraging findings for the efficacy of AO-PTS on children's SEL skills and provided partial support for the two hypotheses in this current study; however, in Analysis 2, when three intervention schools were compared with three-matched control schools, there was no preferential outcomes for the intervention over control schools. In fact, there was a tendency for both groups to improve.

### Behavioral and emotional well-being

Partial support was found for the hypothesis that parents will report lower levels of internalizing and externalizing symptoms and higher levels of total difficulties and prosocial behavior in their children at post-test, compared to pre-test levels; however, this support was only obtained in the primary analysis that comprised of intervention schools—with significant reductions in parent-reported overall Total Difficulties and Hyperactive/Inattention Problems in their children. There was, however, no support with relation to the other subscales of the SDQ-P (Emotion, Conduct, Peer Problems, and Prosocial Behavior).

The finding of reduced total difficulties in the primary analysis of intervention schools is consistent with two studies of Aussie Optimism for older children and of the AO-PTS that also found reduced total difficulties (Swannell et al., 2009; Roberts et al., 2010; Rooney et al., 2013). The finding of reduced hyperactivity in the primary analysis was consistent with Rooney and colleague's findings (Morrison et al., 2013) and findings from the secondary analysis which found no significant difference between intervention and control children on parent reported Hyperactivity, Emotion, Conduct or Peer Relation problems were not consistent with Rooney et al. (2013), Morrison et al. (2013). On the other hand, the finding from the secondary analysis indicating an increase in peer-related problems was not consistent with Lacey et al. (submitted).

The current study findings of no significant improvements in Prosocial Behaviors, Conduct Problems, or Emotion Problems, were inconsistent with other studies which found increases in prosocial behaviors (Payton et al., 2008; Conduct Problems Prevention Research Group, 2010; Durlak et al., 2011; Jones et al., 2011; Schonert-Reichl et al., 2012), reduced conduct problems (Payton et al., 2008; Swannell et al., 2009; Conduct Problems Prevention Research Group, 2010; Durlak et al., 2011; Jones et al., 2011; Schonert-Reichl et al., 2012), and reduced internalizing behaviors (Payton et al., 2008; Durlak et al., 2011). These differences in findings may be related to differences in study sample, measurement instruments, programs, and raters.

The unexpected finding that intervention children significantly increased in parent-reported Peer Problems is not consistent with findings from previous research; for instance, the AO-PTS large scales study found significant decrease in peer-relation problems (Rooney et al., 2013). Furthermore, this finding is in contrast to the enhanced AO-PTS small pilot study which demonstrated a preventive effect on the peer problems (Lacey et al., submitted) and a Strong Start program piloted on one class of students that showed an improvement in Peer Relationships, compared to another class who received mathematics class who worsened in peer relationship (Caldarella et al., 2009). It is difficult to explain why the intervention schools in Analysis 2 would worsen in their parent-reported peer problems. Examination of the children in the secondary analysis of three intervention schools showed that there was a greater proportion (72%) of Year 4's compared to the control schools (51%). Hence, the increased peer problems in the intervention schools may be due to the fact that Year 4s had greater level of peer problems than the Year 5s. Examination of means showed that Year 4 children had consistently more problems and lower levels of prosocial behavior compared to Year 5 s. These differences may indicate developmental differences such that the younger group of children are still learning and consolidating these skills, whereas the older children have had more time to practice.

Unlike the current study, Swannell et al. (2009) found gender differences in the 20 week version of Aussie Optimism, such that males' prosocial behavior worsened, whereas females had significant improvements in their emotion, conduct, and peer problems. In this study, however, gender differences were found across groups with both intervention and control girls having lower levels of total difficulties than both groups of boys.

### Emotion and social knowledge

Partial support was found for the hypothesis that intervention children will report higher levels of emotion attribution accuracy in Analysis 1, with significant increases on the overall emotion attribution accuracy score; on the total emotional attribution of social behaviors and the related subscales happy, mad, and no feelings; and on the total emotional attribution of social situations and the related subscales mad and scared. However, no preferential improvements were found in favor of the intervention children when compared to control school children and thus the intervention effect may be due to extraneous factors.

When considering the primary analysis of intervention schools only, the findings of improvements in emotion knowledge are consistent with those found in one of the PATH's studies (Domitrovich et al., 2007) and in an emotion-based prevention program of 2- to 5-year old children who were reported to have greater increases in emotion knowledge and social competence (Izard et al., 2008). However, the above positive findings did not remain in Analysis 2; and the lack of improvements in children's empathy or perspective taking are consistent with findings in the “roots of empathy” study (Schonert-Reichl et al., 2012).

In the current study gender and age differences were found in both groups. Intervention and control girls had significantly higher emotional attribution accuracy than boys at pre- and post-test. Year 5 students in both groups, had higher total emotion attribution accuracy, compared to Year 4 students.

There are several potential explanations for the age difference findings. According to Piaget's theory, the middle childhood period is a time when children are moving into the concrete operational stage, between the ages of 7 and 12 years; this is a period of time when children start to reason more logically about concrete objects and events (Piaget, 1952). Thus, perhaps the younger students in this study were less well-developed than their Year 5 counterparts. In addition, research on brain development has indicated that important changes take place during middle childhood, with improvements in prefrontal cortex functioning related with planning, decision-making, emotion regulation, and abstract thought (Steinberg, 2005). It is therefore possible that the differential effects found between the Year 4 and 5 children are due to the elder children being more advanced in their brain development and cognitive ability, which may translate to better SEL capability, such as regulating themselves, planning behavior and problem solving in social situations and having better impulse control. In addition, perspective taking skills are also emerging in middle childhood and children during this period are becoming more self-aware, reflective, and less egocentric (Selman, 1980). Emotion knowledge has been shown to be important in children's social and affective development and essential for competence in social situations (Halberstadt et al., 2001; Buckley et al., 2003). Given the importance of emotion knowledge, other modifications to AO-PTS program could include an increased focus on perspective taking and the greater emphasis on recognition of emotions in self and others.

### Explanations for non-significant findings

There are several possible explanations as to why there were no significant findings for an intervention effect when intervention schools were compared to control schools. First, assessment immediately following intervention at post-test may be too early to detect changes in children's SEL skills, as it may take time for children to practice these skills and integrate them into their behavioral repertoire before changes will be noticeable on outcome measures. For instance, other studies have found no intervention effects immediately at post-test, but detect intervention effects at follow-up (Dadds et al., 1997; Quayle et al., 2001; Roberts et al., 2003). For example, this delayed intervention effect was found for Prosocial and Total Difficulties on the SDQ at 12 month follow-up, but not immediately post-intervention, in an evaluation of *Zippy's Friends* in a large scale RCT of 6- to 8-year old primary school children in Ireland (Clarke, 2011). The larger AO-PTS study of 20 schools, from which this data were taken, will be collecting data at 6- and 18-months follow-up period and will thus be able to assess for delayed intervention effects. However, the intervention children would need to show greater improvement in order to rule out maturation effects. Second, control schools may have run other SEL-based programs or have done so in the recent past; such programs would account for the equivalent improvement in control school children emotion knowledge and problems. Future research should attempt to collect information on all current or previous SEL programs in schools. Third, as part of the larger ongoing AO-PTS research, which investigates the effects of the program on anxiety and depression, a report is provided to parents on any children who are assessed as “at risk.” This may have impeded the demonstration of efficacy of the intervention program as it is possible that parents of control group children identified as “at risk” may have sought professional help to assist their children to overcome their problems.

A further possible explanation for the lack of intervention effects in comparison to control schools may be the relatively high baseline levels of emotional knowledge and low levels of emotional and behavioral problems at baseline in this sample, which consisted of children from private schools, when compared to other Aussie Optimism studies (Swannell et al., 2009; Rooney et al., 2013). Previous research has shown that children with higher levels of problems at baseline can benefit more from the intervention (e.g., Swannell et al., 2009). It could be the case that the children in this study were too high functioning to see changes in their scores as there was a ceiling effect in place. It would have been useful to have included an analysis of children at different levels of problems to see if (1) there were any treatment effects (moved into normal range from clinical range) or (2) any preventative effects (no increased levels of problems from normal to clinical range). This type of analysis would have provided a more nuanced view on which type of cases the intervention is effective for, which may be lost in a generalized average of pre- and post-test scores across all children in a school.

Another issue which may have hampered efforts to establish whether or not AO-PTS has positive impacts on children's SEL skills is to do with methods of measurements. First, the ACES in this study had inadequate reliability and was unstable across time—this may have reduced the power of the analyses to detect differences between groups. In addition, the ACES is a measure of only one aspect of SEL, namely social awareness. However, another way of assessing effectiveness of AO-PTS on SEL may have been to investigate whether or not children acquired the SEL skills in the program. For instance, other studies have included a SEL knowledge acquisition assessment (e.g., Strong Start program, Whitcomb and Merrell, 2012). A further change to the way SEL was measured could be to include different SEL measurement tools that tap into more of the five core elements of SEL. There are several recent resources that have listed and summarized the psychometric properties and applications of different SEL measures in children (e.g., Denham et al., 2010; Schonert-Reichl et al., 2010; Haggerty et al., 2011). A further measurement issues is who is rating the SEL skills. Evidence suggests that different raters can provide different ratings. For instance, one study found no change in skills or levels of problems when parents provided ratings of their children, however, significant improvements in children's outcomes were seen when teachers rated the children (Dadds and Roth, 2008). In the current study, levels of problems and prosocial behavior were rated by parents. Parents may have less opportunity to view changes in their children's SEL skills as there is potentially less variety of social interactions in the home environment. Thus, future research should also collect teacher's ratings to reflect changes that may be evident in the school context and provide the opportunity to triangulate outcome data.

The non-significant findings in this study may also have been a result of low implementation quality. Although the program offered five coaching sessions, only small numbers of teachers made use of this opportunity. Unfortunately, these teachers were only coached in one session due to teachers' time constraints and schedule. One limitation of this study was the lack of implementation fidelity assessment; thus, it is not known to what extent the intervention was implemented as planned. Fidelity checks are essential as programs with no implementation problems are associated with better outcomes (Durlak et al., 2011). In future studies of AO-PTS, when recruiting and training school teachers, the importance of implementation fidelity components of the study need to be stressed. One possible strategy to boost the implementation quality is to re-design the teacher's logbook to make it shorter, concise and not to be perceived as extra burden to teachers' current job capacity. It has been found that the teaching context, workload and satisfaction affect teachers' performance in class (Smith and Bourke, 1992).

Another possible limitation of this study is related to teachers' attitudes and beliefs about the effectiveness of the intervention program. Studies have shown that teachers' behavior and teaching practices can be strongly predicted by their attitudes and beliefs (Kagan, 1992; Rimm-Kaufman and Sawyer, 2004). Furthermore, it has been found that teachers' beliefs about social and emotional regulation anticipate teachers' self-awareness and recognition regarding skills practices (Van Ewijk and Van Der Werf, 2012). Students will sustain the new skills learnt and implement these in and outside the classroom if they are taught by teachers who have robust beliefs toward the effectiveness of the intervention program and who have demonstrated high levels of self-efficacy and positive attitudes (Rosen et al., 2009). The Year 4 and 5 teachers involved in the current study were asked to conduct the program by the school principals and attended a 1 day workshop to equip and prepare them on the theories and activities required to run the program in class. Some teachers were not keen to participate and were reluctant about being observed when implementing the program and this may have influenced the effectiveness of study outcomes. A baseline assessment is needed to assess teachers' overall view on the value of mental health prevention programs like the AO-PTS to determine any factors that may promote or hinder program efficacy. However, the drawback in assessing teachers' beliefs and attitudes in the current study is that it may have been perceived as additional workload for the participating teachers as this kind of assessment usually takes the form of a self-report (Van Ewijk and Van Der Werf, 2012). A further issue with self-report of this kind is the potential for teacher bias in responding in favor of the expected results in order of promote mental health programs in the school context.

### Future directions

Many future directions for AO-PTS and other universal SEL program research have already been discussed. Another future direction for SEL program research is to link the acquisition and presence of specific SEL core elements with impacts on specific types of outcomes and for which types of children and cases. This type of information will guide the development and modification of SEL universal program to best target and direct resources into those skills that are linked with positive outcomes. Also, potential age and gender differences highlight the need to include these factors as part of analyses.

## Conclusion

This study investigated the efficacy of a school-based universal program that is delivered by teachers as part of regular class curricula on middle primary school children's SEL skills. This study highlights how different results can be obtained with or without a comparison group, and call into question findings which do not include control groups. At this point in time, this study showed no strong evidence to support the efficacy of AO-PTS on improving SEL skills of Year 4 and 5 children. Methodological issues will need to be addressed in future research. AO-PTS has established evidence for its effectiveness (i) on prevention and treatment of depression symptoms and disorders, (ii) to increase positive attribution, (iii) to reduce the children's total difficulties problems, and (iv) to reduce hyperactive behaviors. However, its effectiveness of SEL skills remains to be established and needs to be investigated further.

### Conflict of interest statement

The authors declare that the research was conducted in the absence of any commercial or financial relationships that could be construed as a potential conflict of interest.
