# Supplementary material for: The efficacy of the enhanced Aussie Optimism Positive Thinking Skills Program in improving social and emotional learning in middle childhood
Source: Front Psychol. 2014 Aug 15;5:909. doi: 10.3389/fpsyg.2014.00909 (PMC4133646; doi:10.3389/fpsyg.2014.00909)
Supplement: Supplementary file 1 [file DataSheet1.ZIP › Appendix (Table 5).DOCX]

Table 5

*Means and Standard Errors for the Parent-Reported Externalising, Internalising, and Prosocial Outcomes in Analysis 1 and Analysis 2 for Intervention and Control School Children*

|  | Analysis 1: Seven Intervention Schools (*N* =386) | | | Analysis 2: Intervention (*N* =164) vs  Control (*N* =107) Schools | | |  |
| --- | --- | --- | --- | --- | --- | --- | --- |
|  |  | Pre-test  *M (SE)* | Post-test  *M (SE)* |  | Pre-test  *M (SE)* | Post-test  *M (SE)* |  |
| Total Difficulties | | Intervention | 7.65 (0.23) | 7.10 (0.37) *^b^* | Intervention  Control | 7.25 (0.33)  8.71 (0.44) | 6.78 (0.58) *^a^*  7.66 (0.21) *^a^* |
| Emotional Symptoms | Intervention | 2.07 (0.07) | 1.87 (0.14) | Intervention  Control | 1.99 (0.13)  2.26 (0.22) | 1.78 (0.24) *^b^*  1.82 (0.22) *^b^* |  |
| Conduct Problems | Intervention | 1.20 (0.05) | 1.12 (0.07) | Intervention  Control | 1.22 (0.08)  1.46 (0.18) | 1.02 (0.12) *^c^*  1.28 (0.14) *^c^* |  |
| Hyperactivity/  Inattentive | Intervention | 3.08 (0.14) | 2.81 (0.13) *^b^* | Intervention  Control | 2.91 (0.16)  3.59 (0.26) | 2.72 (0.20)  3.28 (0.28) |  |
| Peer Problems | Intervention | 1.30 (0.08) | 1.28 (0.09) | Intervention  Control | 1.16 (0.10)  1.41 (0.05) | 1.26 (0.07) *^a^**  1.29 (0.13) *^a^** |  |
| Prosocial Behaviour | Intervention | 8.36 (0.80) | 8.44 (0.08) | Intervention  Control | 8.46 (0.72)  8.27 (0.06) | 8.44 (0.09)  8.43 (0.23) |  |

Note: *^a^* Significant time difference at p<.05; *^b^* Significant time difference at p<.01; *^c^* Significant time difference at p<.001

*Significant interaction at p<.05; **Significant interaction at p<.01; ***Significant interaction at p<.001
