# Supplementary material for: The efficacy of the enhanced Aussie Optimism Positive Thinking Skills Program in improving social and emotional learning in middle childhood
Source: Front Psychol. 2014 Aug 15;5:909. doi: 10.3389/fpsyg.2014.00909 (PMC4133646; doi:10.3389/fpsyg.2014.00909)
Supplement: Supplementary file 1 [file DataSheet1.ZIP › Appendix (Tables 1-4).PDF]

Appendix A

Table 1

Table 1

Summary of Ethnic Origin of Children

Ethnic Origin	Australian	Asian	British Isles	New Zealand	European	Other
**Percentage (%)**	56.2	7.1	21.7	3.9	3.2	8

Table 2

Table 2

Original Number of Students in Each Group by Year Level and Gender

Analysis	Group	Year 4	Year 5	Males	Females	Total
**1.**	**Intervention Schools**	299 (56.4)	231 (43.6%)	260 (49.1%)	270 (50.9%)	530
**2.**	**Intervention Schools**	157 (72%)	61 (28%)	102 (46.8%)	116 (53.2%)	218
	**Control Schools**	80 (51%)	74 (48.1%)	90 (58.8%)	63 (41.2%)	153

Table 3

Table 3

Summary of Positive Thinking Skills Program

Module	Topic	Aim of Module
1	Introduction and Planning for fun activities	Orienting the students to the program and for students to list activities they enjoy and understand the importance of doing these things regularly.
2	Identifying my feelings	For students to identify a range of feelings
3	Comfortable and uncomfortable feelings	For students to learn to notice body clues for comfortable and uncomfortable feelings.
4	Feelings and situations	For students to understand the link between situations and feelings and how situations can affect how we feel and behave.

5	Catching your thoughts	For students to understand the link between thoughts and feelings and to tune into their “self-talk”.
6	Being brave	For students to learn the skills to overcome fears and challenging situations.
7	Thought-feeling connection	For students to understand the link between thought, feelings, and behaviour and to begin evaluating thoughts to see how realist they are.
8	Helpful and unhelpful thinking	For students to become familiar with the types and impact of unhelpful thinking.
9	Looking for evidence to change your thoughts	For student to look for evidence to be able to generate alternative thoughts.
10	Self–esteem and being brave	For students to build their self-esteem though sharing how they went with their BRAVE staircase and revision of the AO-PTS content.

Table 4

Table 4

Means and Standard Errors for the Child-Reported Outcomes in Analysis 1 and Analysis 2 for Intervention and Control School Children

	Analysis 1: Seven Intervention Schools			Analysis 2: Intervention vs Control Schools		
		Pretest *M (SE)*	Posttest *M (SE)*		Pretest *M (SE)*	Posttest *M (SE)*
**EAS**	Intervention	19.07(0.34)	19.91 (0.25)[c]	Intervention<br>Control	19.42 (0.34)<br>19.07 (0.52)	20.18 (0.19)[c]<br>20.07 (0.39)[c]
**BS Total**	Intervention	9.39 (0.18)	9.81 (0.15)[c]	Intervention<br>Control	9.89 (0.13)<br>9.74 (0.27)	10.25 (0.12)[b]<br>10.23 (0.38)[b]
**BS Happy**	Intervention	2.70 (0.04)	2.77 (0.02)[b]	Intervention<br>Control	2.75 (0.04)<br>2.79 (0.05)	2.81 (0.10)<br>2.42 (0.02)
**BS Sad**	Intervention	2.54 (0.02)	2.60 (0.04)	Intervention<br>Control	2.56 (0.02)<br>2.42 (0.02)	2.62 (0.01)[c]***<br>2.61 (0.06) ***
**BS Mad**	Intervention	2.12 (0.08)	2.36 (0.07)	Intervention<br>Control	2.14 (0.08)<br>2.14 (0.09)	2.31 (0.10)[b]<br>2.28 (0.11)[b]
**BS Scared**	Intervention	2.04 (0.07)	2.09 (0.06)	Intervention<br>Control	.07 (0.11)<br>1.99 (0.13)	2.21 (0.07)[c]<br>2.18 (0.16)[c]
**BS Ambig**	Intervention	0.34 (0.03)	1.03 (0.01)[c]	Intervention<br>Control	0.30 (0.04)<br>0.35 (0.34)	1.02 (0.02)[c]<br>1.14 (0.04)[c]
**SS Total**	Intervention	9.68 (0.17)	10.12 (0.12)[c]	Intervention<br>Control	9.89 (0.13)<br>9.74 (0.27)	10.25 (0.12)[b]<br>10.23 (0.04)[b]
**SS Happy**	Intervention	2.81 (0.03)	2.84 (0.02)	Intervention<br>Control	2.83 (0.02)<br>2.78 (0.05)	2.87 (0.00)[c]<br>2.85 (0.02)
**SS Sad**	Intervention	2.72 (0.04)	2.76 (0.03)	Intervention<br>Control	2.79 (0.03)<br>2.74 (0.07)	2.78 (0.05)[c]<br>2.83 (0.03)
**SS Mad**	Intervention	1.83 (0.05)	2.01 (0.06)[b]	Intervention<br>Control	1.89 (0.02)<br>1.87 (0.11)	2.05 (0.11)<br>2.02 (0.08)
**SS Scared**	Intervention	2.31 (0.09)	2.51 (0.06)[c]	Intervention<br>Control	2.38 (0.12)<br>2.35 (0.07)	2.56 (0.06)[c]<br>2.54 (0.03)[c]
**SS Ambig**	Intervention	0.04 (0.01)	0.04 (0.01)	Intervention<br>Control	0.05 (0.03)<br>0.08 (0.03)	0.03 (0.02)<br>0.05 (0.02)

Note: ACES=The Assessment of Children's Emotional Skills; EAS=Total Emotion Attribution Score; BSTotal=Total Behavioural Situations score; BSHappy= Happy Behavioural Situations; BSSad=Sad Behavioural Situations; BSMad=Mad Behavioural Situations; BSScared= Scared Behavioural Situations; BSAmbig= Ambiguous Behavioural Situations; SSTotal=Total Social Situations score; SSHappy= Happiness Social Situations; BSSad=Sad Social Situations; BSMad= Mad Social Situations; BSScared= Scared Social Situations; BSAmbig= Ambiguous Social Situations.

[a] Significant time difference at p<.05; [b] Significant time difference at p<.01; [c] Significant time difference at p<.001*Significant interaction at p<.05; **Significant interaction at p<.01; ***Significant interaction at p<.001
